# Atrial AMP-activated protein kinase is critical for prevention of dysregulation of electrical excitability and atrial fibrillation

**DOI:** 10.1172/jci.insight.141213

**Published:** 2022-04-22

**Authors:** Kevin N. Su, Yina Ma, Marine Cacheux, Zeki Ilkan, Nour Raad, Grace K. Muller, Xiaohong Wu, Nicole Guerrera, Stephanie L. Thorn, Albert J. Sinusas, Marc Foretz, Benoit Viollet, Joseph G. Akar, Fadi G. Akar, Lawrence H. Young

**Affiliations:** 1 Department of Cellular & Molecular Physiology and; 2 Department of Internal Medicine, Yale Cardiovascular Research Center, Yale University School of Medicine, New Haven, Connecticut, USA.; 3 Cardiovascular Institute, Icahn School of Medicine at Mount Sinai, New York, New York, USA.; 4 Department of Radiology and Biomedical Imaging, Yale University School of Medicine, New Haven, Connecticut, USA.; 5 Institut Cochin, Université de Paris, CNRS, INSERM, Paris, France.

**Keywords:** Cardiology, Arrhythmias, Protein kinases

## Abstract

Metabolic stress is an important cause of pathological atrial remodeling and atrial fibrillation. AMPK is a ubiquitous master metabolic regulator, yet its biological function in the atria is poorly understood in both health and disease. We investigated the impact of atrium-selective cardiac AMPK deletion on electrophysiological and structural remodeling in mice. Loss of atrial AMPK expression caused atrial changes in electrophysiological properties and atrial ectopic activity prior to the onset of spontaneous atrial fibrillation. Concomitant transcriptional downregulation of connexins and atrial ion channel subunits manifested with delayed left atrial activation and repolarization. The early molecular and electrophysiological abnormalities preceded left atrial structural remodeling and interstitial fibrosis. AMPK inactivation induced downregulation of transcription factors (*Mef2c* and *Pitx2c*) linked to connexin and ion channel transcriptional reprogramming. Thus, AMPK plays an essential homeostatic role in atria, protecting against adverse remodeling potentially by regulating key transcription factors that control the expression of atrial ion channels and gap junction proteins.

## Introduction

Atrial fibrillation is the most prevalent cardiac arrhythmia and constitutes a major risk factor for stroke, heart failure, and dementia (1, 2). There is growing evidence that links atrial fibrillation to metabolic stress and inflammation (3). Although atrial fibrillation is common with aging and in prevalent metabolic disorders, such as diabetes and obesity (4), the mechanisms underlying the development and perpetuation of atrial fibrillation during metabolic stress are poorly defined.

The pathophysiology of atrial fibrillation involves progressive electrical and structural remodeling that underlie the progression of the arrhythmia from paroxysmal to a more persistent form. The remodeling of ion channels and gap junction proteins leads to abnormal automaticity, triggered activity, and spatiotemporal changes in atrial conduction and repolarization properties (5). Atrial dilation and interstitial fibrosis, which develop as the consequences of increased wall tension, inflammation, and oxidative stress (4, 6), also promote conduction block and electrical reentry. In combination, atrial electrical and structural remodeling promote the triggers and substrate leading to the onset and maintenance of atrial fibrillation (5, 7).

Human genome-wide association studies have identified susceptibility loci in the genes encoding important determinants of these processes, including *KNCJ2*, *SCN5A*, *GJA5*, *GATA4*, and *NKX2-5* (8–10). Although genetic predisposition accounts for a small subset of patients with atrial fibrillation, the molecular mechanisms underlying the more common acquired forms remain uncertain. AMPK is a ubiquitously expressed serine/threonine kinase that is a critical regulator of cellular metabolism (11). Activation of the AMPK pathway plays an important role in the myocardial response to ischemia (12, 13), pressure overload (14, 15), and heart failure (16, 17). When activated, AMPK orchestrates a coordinated cellular response that promotes fatty acid oxidation (12) and stimulates glucose transport (18) and glycolysis (19) while conserving energy stores (13). Although previous studies have elucidated the actions of AMPK in the left ventricle, the biological effects of endogenous AMPK activation in the atria are poorly understood. Activated AMPK regulates cellular calcium handling and contraction during acute metabolic inhibition of atrial cardiomyocytes (20), but its role in maintaining atrial electrical homeostasis and structural integrity in vivo is uncertain.

Given these observations and the growing recognition of the impact of metabolic disease in the genesis of atrial fibrillation, the aim of this study was to define the role of AMPK in atrial molecular, electrophysiological, and structural remodeling. We generated a mouse line with atrium-selective genetic depletion of AMPK to study its autonomous function in the atria. We report that these mice demonstrated a hierarchy of pathological events, including initial atrial electrophysiological derangements followed by structural remodeling and fibrosis that culminated in the spontaneous onset of sustained atrial fibrillation. We further identified *Pitx2c* and *Mef2c* as transcription factors that are dysregulated in the context of AMPK depletion and control the expression of genes that are critical in maintaining atrial electrical homeostasis.

## Results

### Atrial AMPK deletion and downstream pathway inactivation.

**Figure 1:**
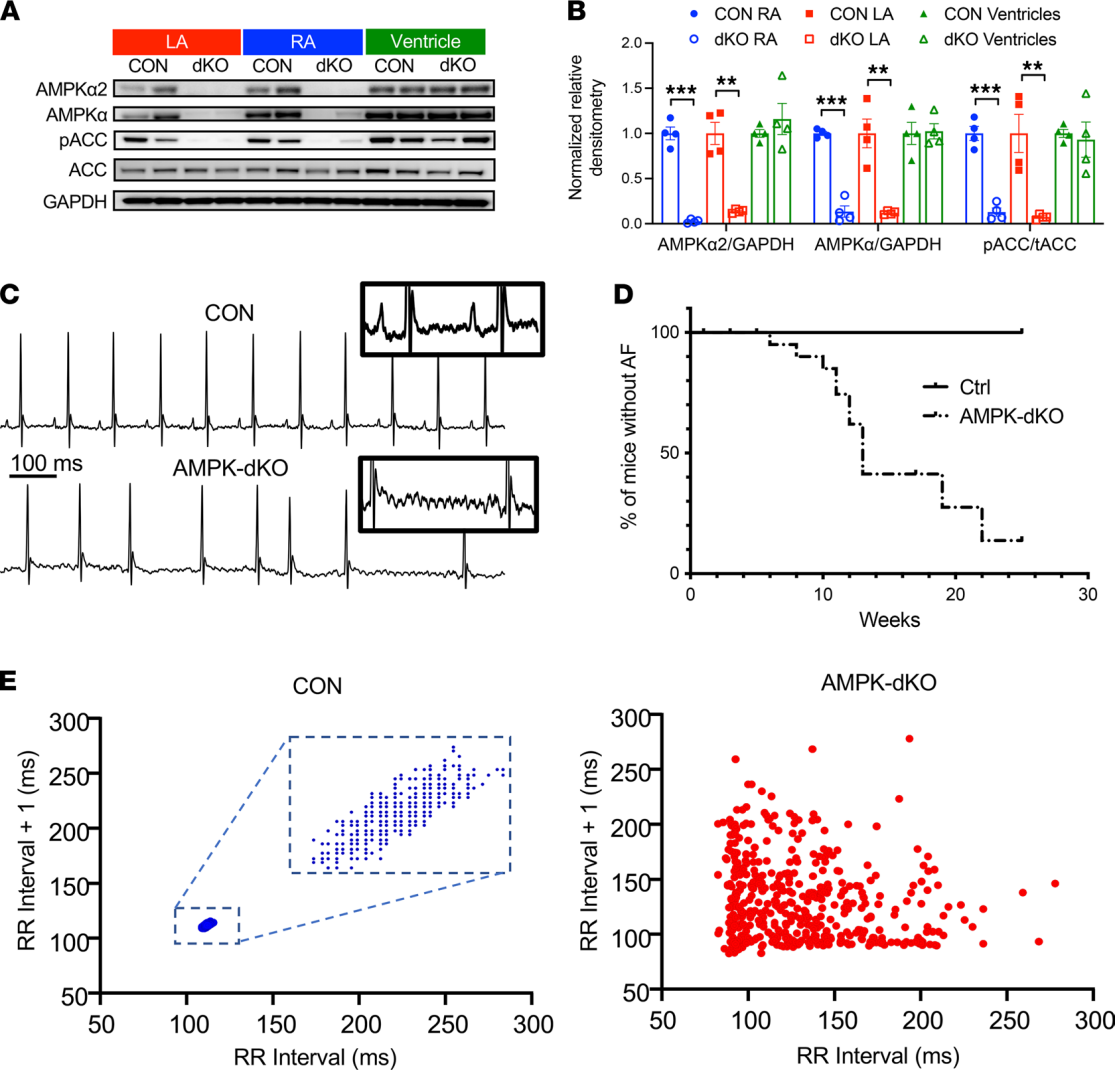
Atrium-selective deletion of AMPK leads to atrial fibrillation in mice. AMPK expression and the development of atrial fibrillation in AMPK double-KO *sarcolipin-Cre Prkaa1^fl/fl^ Prkaa2^fl/fl^* (AMPK-dKO) compared with littermate control *Prkaa1^fl/fl^ Prkaa2^fl/fl^* (CON) mice. (**A**) Representative immunoblots with antibodies recognizing the AMPK α2 subunit, both α1 and α2 AMPK subunits (AMPKα), the downstream AMPK target acetyl-CoA carboxylase (ACC), and the phosphorylated form of ACC (pACC) in the left atria (LA), right atria (RA), and left ventricle. (**B**) Corresponding densitometric quantification of the immunoreactive bands. Values are mean ± SEM of *n* = 6–8 per group. **P < 0.01, ***P < 0.001 versus CON by unpaired Student’s *t* test. (**C**) Representative in vivo ECG (lead II) tracings showing normal sinus rhythm in a CON and atrial fibrillation in an atrial AMPK-dKO mouse at 6 weeks of age. Insets show magnified views that demonstrate highly organized atrial P-wave electrical activity in CON and course fibrillatory waves in AMPK-dKO mice. Scale bars: 100 ms. (**D**) Kaplan-Meier analysis showing the 6-month event-free survival from atrial fibrillation in CON (*n* = 18) versus AMPK-dKO mice (*n* = 23), *P* < 0.001. (**E**) Graphs show heart rate variability in representative examples of CON mice in sinus rhythm and AMPK-dKO mice in atrial fibrillation, comparing sequential R-R intervals during 60-second recordings (*n* = 533 intervals for CON and *n* = 454 intervals for AMPK-dKO mice).

To study the effects of atrial AMPK pathway inactivation on atrial structural and electrical remodeling, we generated mice with conditional deletion of AMPK α1 (*Prkaa1*) and α2 (*Prkaa2*) catalytic subunits in the atria, using the sarcolipin (Sln) promoter to drive Cre-recombinase expression. The *Sln-Cre Prkaa1^fl/fl^ Prkaa2^fl/fl^* mice, referred to herein as atrial AMPK double-KO (AMPK-dKO), demonstrated more than 90% reduction in the expression of AMPK α subunits on immunoblots of both left and right atrial homogenates (Figure 1, A and B). There was also evidence of markedly diminished phosphorylation of the AMPK downstream target, acetyl-CoA carboxylase (ACC), compared with littermate *Prkaa1^fl/fl^ Prkaa2^fl/fl^* control mice (Figure 1, A and B). The cardiac deletion was highly selective to the atria, with the atrial AMPK-dKO mice showing no change in ventricular AMPK α subunit expression or downstream pathway activation (Figure 1, A and B). Furthermore, the degree of AMPK depletion was comparable in the right and left atria from the atrial AMPK-dKO mice (Figure 1, A and B).

### Atrial AMPK depletion causes spontaneous atrial fibrillation in AMPK-dKO mice.

In order to assess the electrophysiological consequences of atrial AMPK depletion in vivo, we first performed serial intermittent ECG recordings in control and atrial AMPK-dKO mice between the ages of 1 week and 6 months. Control mice demonstrated normal sinus rhythm throughout the entire study with no evidence of atrial arrhythmia (Figure 1, C and D). The atrial AMPK-dKO mice all displayed sinus rhythm after birth, but they began to demonstrate spontaneous atrial fibrillation as early as 6 weeks of age (Figure 1, C and D). These episodes were characterized by highly disorganized and rapid atrial electrical activity associated with an irregular ventricular response (Figure 1C). The atrial AMPK-dKO mice showed markedly abnormal beat-beat heart rate variability characteristic of atrial fibrillation, in contrast to control mice that demonstrated minor physiological variability (Figure 1E). At 3 and 6 months of age, spontaneous atrial fibrillation was detected in 50% and 90% of the atrial AMPK-dKO mice, respectively (Figure 1D). Additional control mice expressing Sln-Cre alone (*Sln-Cre Prkaa1^+/+^ Prkaa2^+/+^*) showed no abnormalities on ECG or evidence of atrial fibrillation (Supplemental Figure 1; supplemental material available online with this article; https://doi.org/10.1172/jci.insight.141213DS1).

### Atrial structural remodeling in AMPK-dKO and control mice.

**Figure 2:**
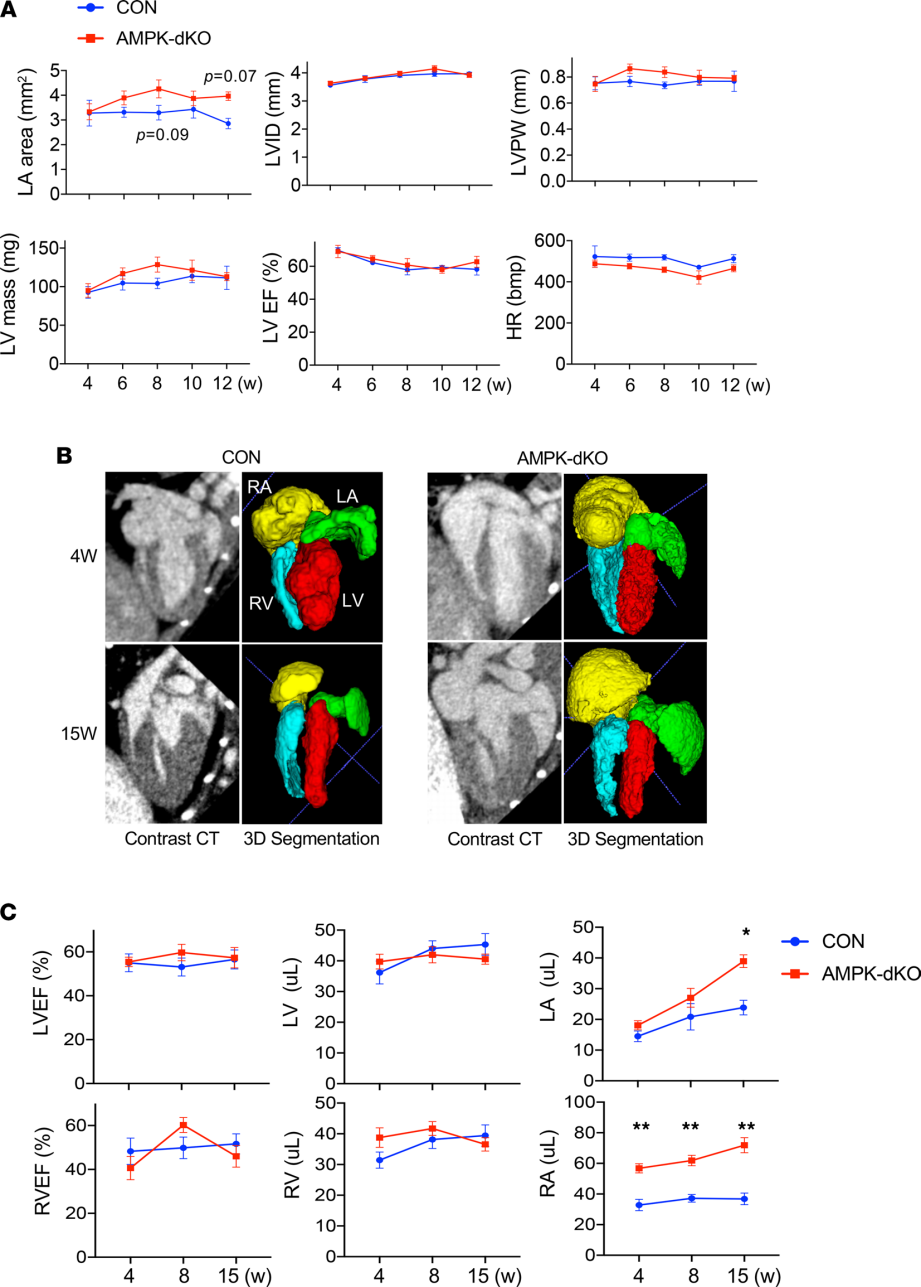
Cardiac structural remodeling in mice with atrial AMPK deletion. Echocardiogram and CT parameters were analyzed in AMPK double-KO *sarcolipin-Cre Prkaa1^fl/fl^ Prkaa2^fl/fl^* (AMPK-dKO) and littermate control *Prkaa1^fl/fl^ Prkaa2^fl/fl^* (CON) mice. (**A**) Serial echocardiographic measurements were performed between 4 and 12 weeks of age; graphs show left atrial (LA) area, left ventricular inner diameter at end-diastole (LVID), LV posterior wall thickness (LVPW), LV ejection fraction (LVEF), and heart rate (HR). Values are mean ± SEM, *n* = 6–9 per group. (**B**) Serial cardiac in vivo micro-CT imaging with contrast enhancement performed between 4 and 15 weeks of age. In representative examples of hearts of 4- and 15-week-old mice, the left panels show grayscale horizontal long axis CT cardiac images, and the right panels demonstrate color 3D segmentation reconstructions of the hearts. CT image acquisition was gated to the ECG cycle and was segmented into 8 phases. Images from atrial diastole (LV end-systole) are shown to visualize the fully filled atria. (**C**) Quantification of cardiac chamber volumes and ventricular ejection fractions (LVEF and RVEF) in mice imaged serially at 4, 8, and 15 weeks of age. Volumes in the fully filled atria (end of LV systole) and ventricles (end of LV diastole) are shown in the graphs. Values are mean ± SEM of *n* = 9–11 per group. *P < 0.05, **P < 0.01 versus matched controls by 2-way ANOVA with Holm-Šidák multiple-comparison test.

Atrial enlargement and interstitial fibrosis are characteristic features of atrial structural remodeling and constitute important contributors to atrial conduction abnormalities and atrial fibrillation (21, 22). We initially assessed cardiac chamber dimensions using serial echocardiography in atrial AMPK-dKO and control mice biweekly from 4 to 12 weeks of age. Left ventricle size, wall thickness, and contractile function (as assessed by ejection fraction) were similar in both groups, with numerical trends toward increased left atrial size in the atrial AMPK-dKO mice at 8 and 12 weeks of age (Figure 2A).

Since echocardiography provides a limited assessment of the left atrium and the right-sided cardiac chambers in mice, we studied a separate cohort of atrial AMPK-dKO and control mice using 3D micro-CT imaging with contrast enhancement. Imaging was performed at 4, 8, and 15 weeks of age to better assess the time-dependent effects of AMPK deletion on cardiac remodeling before and after the onset of atrial fibrillation. CT imaging provided high-resolution images of cardiac chambers (Figure 2B). Integration of multiple slices in 3 orthogonal planes enabled us to perform reliable 3D segmentation in order to accurately quantify chamber volumes.

Micro CT images demonstrated similar left and right ventricular contractile function (ejection fractions) and volumes in atrial AMPK-dKO and control mice (Figure 2, B and C). Although the atrial AMPK-dKO mice had normal left atrial volumes at 4 weeks, they developed progressive left atrial enlargement over time, with 2-fold greater size compared with controls at 15 weeks of age. In contrast, right atrial enlargement developed earlier in the atrial AMPK-dKO mice and was clearly evident by 4 weeks of age. Thus, in the context of atrial AMPK deletion, there was a greater predisposition to early right versus left atrial dilatation before atrial fibrillation onset, with the emergence of biatrial enlargement after the development of the arrhythmia.

**Figure 3:**
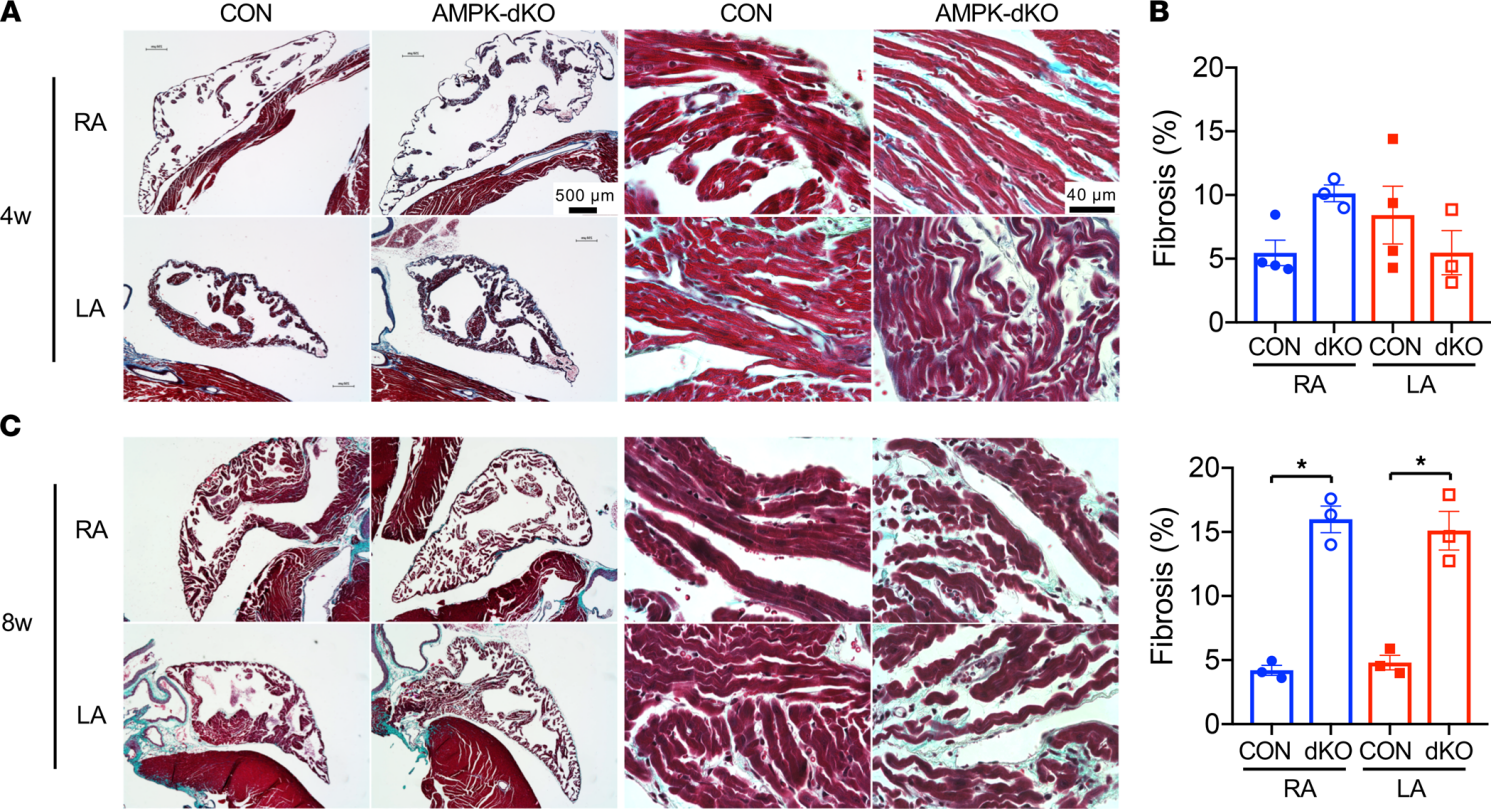
Differential atrial remodeling in mice with atrial AMPK deletion. Cardiac histology was analyzed in AMPK double-KO *sarcolipin-Cre Prkaa1^fl/fl^ Prkaa2^fl/fl^* (AMPK-dKO) and littermate control *Prkaa1^fl/fl^ Prkaa2^fl/fl^* (CON) mice. Representative histological fixed sections of atrial tissues stained with Masson’s trichrome. (**A**) Low-power cross-sectional images of RA and LA chambers (scale bars: 500 μm, first 2 columns) and corresponding higher magnification images of atrial myocardial tissue (scale bars: 40 μm, last 2 columns) from mice at 4 weeks of age. (**B**) Quantification of fibrosis as a percentage of total myocardial tissue area. *n* = 3–4 per group. (**C**) Low-power cross-sectional images of RA and LA chambers (scale bars: 500 μm, first 2 columns) and corresponding higher magnification images of atrial myocardial tissue (scale bars: 40 μm, last 2 columns) from mice at 8 weeks of age. Values are mean ± SEM of *n* = 3 per group. *P < 0.05 versus CON tissues by 1-way ANOVA.

To further assess the link between atrial AMPK deletion and fibrosis in this model, we performed histological analyses on atrial tissue samples from AMPK-dKO and control mice at 4 and 8 weeks of age. There was no evidence of significantly increased interstitial fibrosis in Masson’s trichrome–stained atrial sections from atrial AMPK-dKO mice compared to control mice at 4 weeks of age (Figure 3). There was also no increase in collagen content based on the biochemical analysis of hydroxyproline content in hydrolyzed atrial tissue (Supplemental Figure 2). In contrast, histological analysis of mice at 8 weeks of age demonstrated clear interstitial fibrosis in both the right and left atria of AMPK-dKO compared with control mice (Figure 3), correlating with the time at which they started to develop atrial fibrillation.

### Atrial AMPK-dKO mice display early ECG abnormalities preceding structural remodeling and atrial fibrillation.

**Figure 4:**
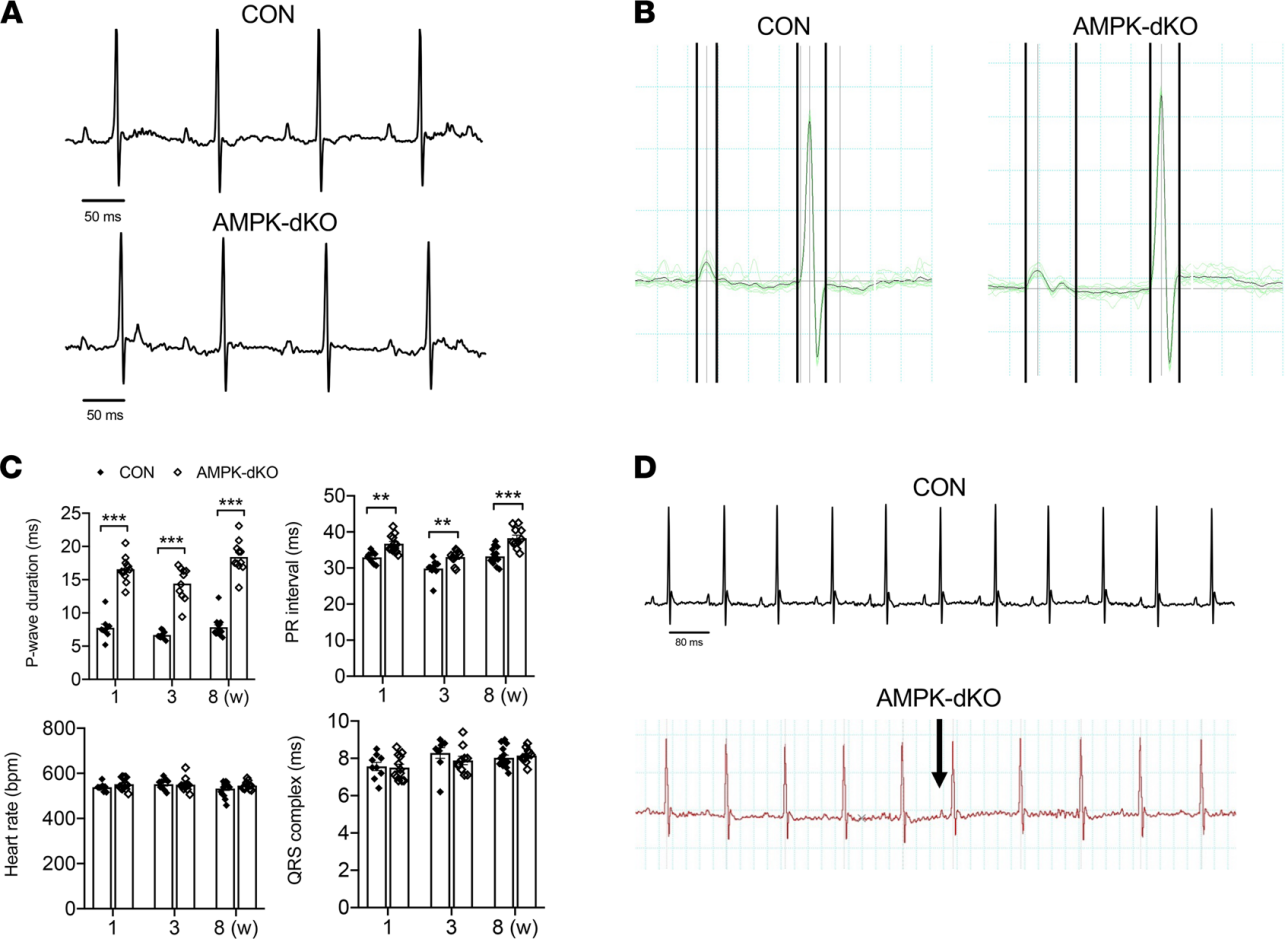
Atrial AMPK deletion prolongs atrial depolarization and causes premature atrial ectopic complexes prior to the development of atrial fibrillation in mice. Serial cardiac in vivo surface ECGs were performed in AMPK double-KO *sarcolipin-Cre Prkaa1^fl/fl^ Prkaa2^fl/fl^* (AMPK-dKO) and littermate control *Prkaa1^fl/fl^ Prkaa2^fl/fl^* (CON) mice. AMPK-dKO mice developed prolonged P-wave duration at a young age without altered ventricular conduction time or heart rate. (**A**) Representative ECGs (lead II) showing P-wave morphology in AMPK-dKO and CON mice at 1 week of age. (**B**) Representative signal-averaged tracings of 10 consecutive heartbeats used for quantitative electrocardiographic analysis. Vertical black lines denote the start and termination of atrial depolarization (P-wave duration) and ventricular depolarization (QRS complex duration). Scale bars 50 ms. (**C**) Quantification of P-wave duration, atrial-ventricular conduction time (PR interval), heart rate (HR), and QRS complex duration in mice at 1, 3, and 8 weeks of age. Values are mean ± SEM of *n* = 6–8. **P < 0.05, ***P < 0.01 versus CON by unpaired Student’s *t* test with correction for multiple comparisons. (**D**) Representative example of ectopic premature atrial complex in an AMPK-dKO mouse (denoted by the black arrow). Scale bar: 80 ms.

To determine the sequence of events by which AMPK deletion culminates in spontaneous atrial fibrillation, we next focused on defining the early ECG signature of this model. While in sinus rhythm prior to the onset of atrial fibrillation, atrial AMPK-dKO mice typically demonstrated fragmented, double-peaked P-waves characteristic of atrial conduction delay (Figure 4, A and B). As early as 1 week of age, there was a 2-fold increase in P-wave duration in atrial AMPK-dKO mice compared with control mice (Figure 4C). The atrial AMPK-dKO mice also exhibited minor prolongation (10%) of their PR intervals, which reflected the sum of intraatrial conduction and AV nodal conduction times (Figure 4C). At this age, all atrial AMPK-dKO and control mice were in sinus rhythm and exhibited comparable heart rates that were within the physiological range under light isoflurane anesthesia. Consistent with the selective atrial deletion of AMPK in the heart, the QRS complex durations, reflecting ventricular activation, were similar in the atrial AMPK-dKO and control mice (Figure 4C). Additional control mice expressing Sln-Cre alone showed normal P-wave duration, PR intervals, and QRS durations (Supplemental Figure 1).

We observed in vivo atrial ectopy in the form of spontaneous premature atrial complexes in atrial AMPK-dKO but not in control mice starting at 2 weeks of age (Figure 4D). The atrial ectopy resulted in pronounced heart rate variability in the atrial AMPK-dKO mice that was manifest in a 3-fold increase in the standard deviation of the heart rate from 2 to 4 weeks of age (Supplemental Figure 3). Thus, significant atrial ectopy, which is an important trigger for atrial fibrillation (23), preceded the onset of atrial fibrillation in this model.

### AMPK deletion causes dysregulation of connexins and ion channels and prolongs atrial conduction.

**Figure 5:**
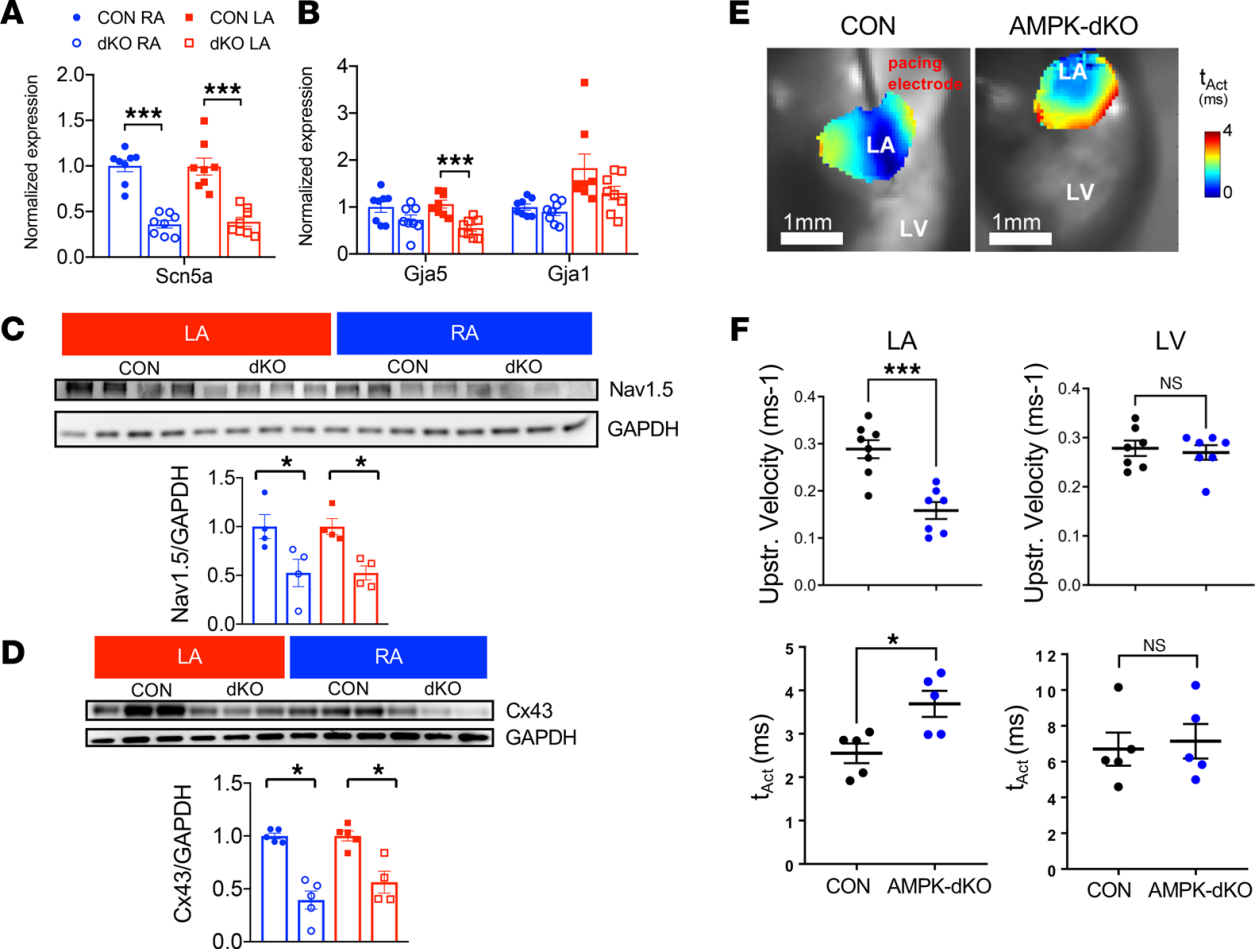
Molecular alterations associated with prolonged left atrial activation time in mice with atrial AMPK deletion. Atrial expression of ion channels and gap junction proteins critical to atrial conduction and ex vivo atrial conduction time in AMPK double-KO *sarcolipin-Cre Prkaa1^fl/fl^ Prkaa2^fl/fl^* (AMPK-dKO) and littermate control *Prkaa1^fl/fl^ Prkaa2^fl/fl^* (CON) mice. (**A**) *Scn5a* mRNA transcripts encoding the voltage-gated sodium channel Nav1.5 and (**B**) *Gja5* and *Gja1* mRNA transcripts encoding the gap junction proteins connexin-40 and connexin-43, respectively, in right atria (RA) and left atria (LA) from 1-week-old mice. (**C**) Representative Nav1.5 immunoblots from RA and LA from 4-week-old mice with corresponding densitometric quantification. GAPDH was used as loading control. Lysates were run on parallel gels simultaneously and probed for total proteins. (**D**) Representative connexin-43 immunoblots from RA and LA from 4-week-old mice with corresponding densitometric quantification. (**E**) Representative left atrial depolarization isochrones from high-resolution optical action potential mapping in CON and AMPK-dKO hearts. Scale bar: 1 mm. (**F**) Quantification of upstroke (upstr.) velocity and tissue activation time (t_Act_) in left atria and left ventricles of 6-week-old mice. Values are mean ± SEM of *n* = 5–8. *P < 0.05, ***P < 0.001 versus control by unpaired Student’s *t* test.

We hypothesized that the prolonged P-wave duration and the propensity to develop atrial fibrillation reflected changes in the molecular determinants of excitability and conduction in the atrial AMPK-dKO mice. Thus, we assessed the atrial expression of gap junction proteins and selected ion channel subunits in the atrial AMPK-dKO and control mice. The atrial AMPK-dKO mice exhibited significant reductions in the right and left atrial levels of *Scn5a* mRNA transcripts, with a corresponding decrease in the expression of Nav1.5 protein, which is the pore-forming subunit of the voltage-gated sodium channel responsible for phase 0 depolarization in cardiomyocytes (Figure 5, A and C). We also found downregulation of atrial connexins in the atrial AMPK-dKO mice. The left atrial level of *Gja5* mRNA transcripts was significantly reduced, and there was a numerical trend to decreased expression of *Gja1* in the atrial AMPK-dKO mice (Figure 5A). The expression of connexin-43 protein encoded by *Gja1* was consistently reduced in both the left and right atria from atrial AMPK-dKO compared with control mice (Figure 5, B and D). There was no change in the expression of connexin-40 protein encoded by *Gja5* (Supplemental Figure 4), suggesting the possibility of altered stability of connexin-40 protein in this model. Thus, important molecules that regulate atrial cardiomyocyte excitability and conduction were perturbed in the atrial AMPK-dKO mice.

In order to determine the electrophysiological impact of these molecular alterations, high-resolution optical action potential mapping was used to directly quantify differences in atrial conduction delay. Isolated perfused hearts were loaded with the voltage-sensitive dye di-4-ANEPPS and subjected to steady-state atrial pacing ex vivo. A charge-coupled device camera was positioned to optimize imaging of the left-sided cardiac chambers. Since the left atrium is the predominant site of origin of most clinical atrial fibrillation, we prioritized studying the left atrium. The mapping studies were performed as soon as technically feasible in young mice so that their interpretation would not be confounded by structural or electrical alterations secondary to the development of atrial fibrillation. Analysis of the mapping studies revealed a 50% decrease in the left atrial upstroke velocity and a 40% increase in left atrial tissue activation times in the atrial AMPK-dKO compared with control mice at 6 weeks of age (Figure 5, E and F). These electrophysiological results indicate that AMPK deletion caused substantial left atrial conduction delay as a primary event prior to the appearance of left atrial structural remodeling or fibrosis.

### AMPK deletion leads to atrial action potential prolongation and downregulation of potassium channels responsible for repolarization.

**Figure 6:**
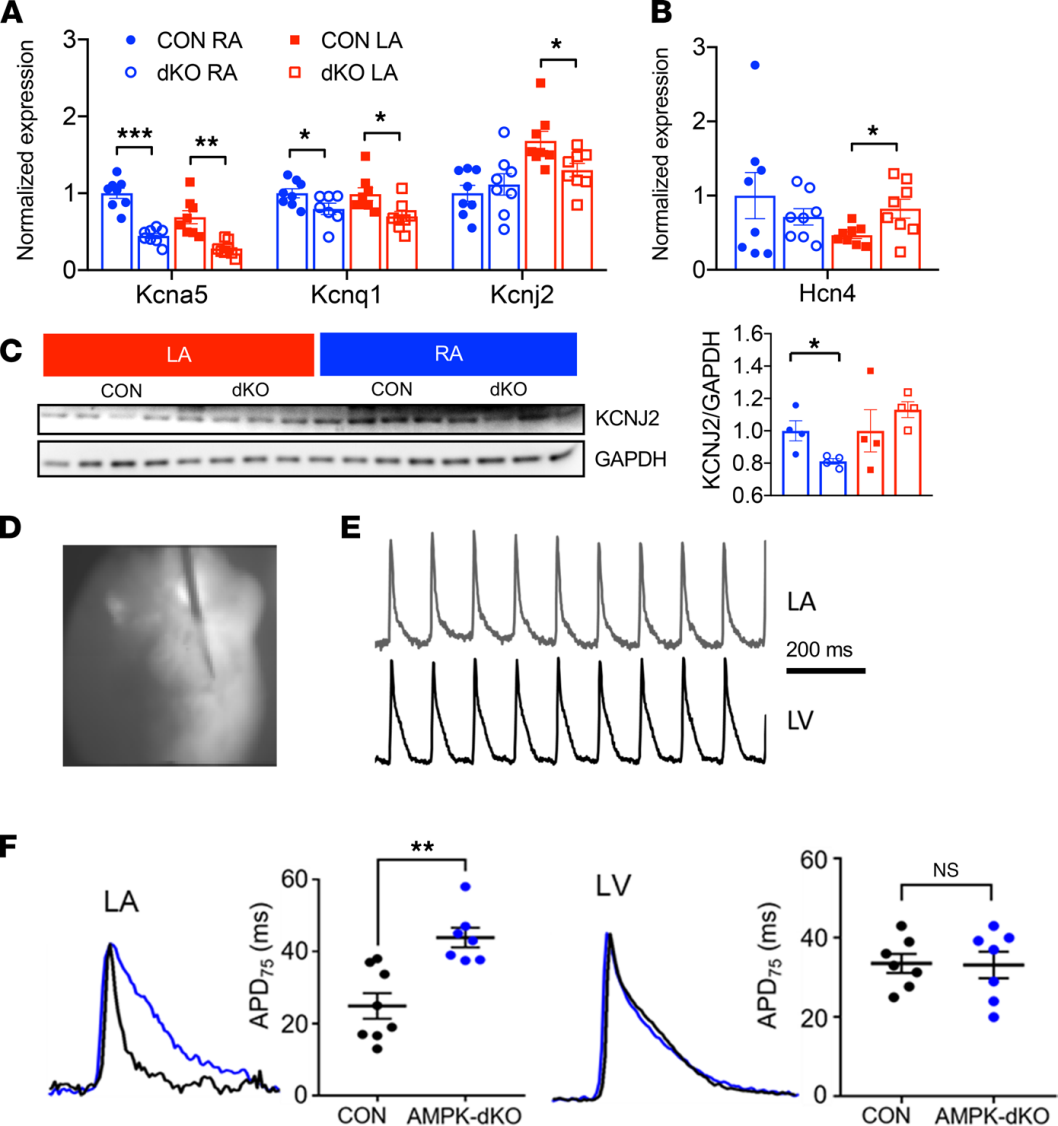
Decreased atrial potassium channel expression and prolonged electrophysiological repolarization in hearts from mice with atrial AMPK deletion. Atrial channel expression and high-resolution optical action potentials in AMPK double-KO *sarcolipin-Cre Prkaa1^fl/fl^ Prkaa2^fl/fll^* (AMPK-dKO) and littermate control *Prkaa1^fl/fl^ Prkaa2^fl/fl^* (CON) mice. (**A**) mRNA transcripts encoding the repolarizing Kv1.5 (*Kcna5*), Kv7.1 (*Kcnq1*), and the inward rectifier Kir2.1 (*Kcnj2*) channels in right atria (RA) and left atria (LA) from 1-week-old mice. (**B**) mRNA transcripts encoding the “funny current” (*Hcn4*) from 1-week-old mice. Values are mean ± SEM of *n* = 6 per group. (**C**) Representative Kir2.1 immunoblots from RA and LA from 4-week-old mice with corresponding densitometric quantification. Proteins were immunoblotted after transfer from the same gel used for Nav1.5 and GAPDH detection (in Figure 5C) and the GAPDH immunoblot was again used as the loading control for Kir2.1. Values are mean ± SEM of *n* = 4 per group. (**D**) Representative image of an ex vivo perfused heart from a 6-week-old CON mouse oriented to optimize the simultaneous mapping of the left atrial and ventricular surfaces during atrial pacing. (**E**) Representative LA and left ventricular (LV) optical action potential traces that document the ability to accurately resolve both depolarization and repolarization. Scale bar: 200 ms. (**F**) Quantification of LA and LV action potential duration (APD_75_) in hearts from 6-week-old mice. Values are mean ± SEM of *n* = 6–8 per group. *P < 0.05, **P < 0.01, ***P < 0.001 versus CON by unpaired Student’s *t* test.

Atrial deletion of AMPK altered the expression of several potassium channels (Figure 6A). Cardiac potassium channels are important determinants of cardiomyocyte repolarization and hence action potential duration. *Kcnq1*, which encodes the pore-forming α subunit Kv7.1, was downregulated in the right and left atria of atrial AMPK-dKO compared with control mice. *Kcna5*, which encodes the voltage-gated potassium channel subunit Kv1.5, was also downregulated in both atria from atrial AMPK-dKO mice. Intriguingly, the expression of the inward rectifier Kir2.1 encoded by *Kcnj2* was differentially higher in the left than right atria at baseline, and AMPK deletion appeared to reduce the left atrial content of both its mRNA level and protein (Figure 6, A and C). Kir2.1 is an important determinant of resting membrane potential, and its downregulation could plausibly contribute to the atrial ectopic activity that was observed in atrial AMPK-dKO mice.

An important determinant of the spontaneous membrane depolarization in nodal cells, the hyperpolarization-activated cyclic nucleotide-gated channel 4 (*Hcn4*), contributes prominently to the so-called pacemaker or funny current (*I*_f_). *Hcn4* expression was significantly higher in the right compared with left atria of control mice, as anticipated based on the anatomic location of the sinoatrial node within the right atrium. Interestingly, *Hcn4* mRNA transcript expression was increased in the left atria of the AMPK-dKO mice, eliminating the differential expression across the 2 atria (Figure 6B). The expression of Hcn4 protein was not reliably detected with available antibodies. However, the upregulation of *Hcn4* in the left atria, particularly in the context of Kir2.1 downregulation, could also potentially contribute to the ectopic atrial activity observed in the atrial AMPK-dKO mice prior to the development of atrial fibrillation.

In order to assess the electrophysiological impact of altered potassium channel expression, we further utilized high-resolution optical action potential mapping to measure the left atrial and ventricular action potential durations. An image of the mapping field of view along with representative left atrial and left ventricular action potential traces are shown in Figure 6, D and E. Quantitative analysis of the action potential durations demonstrated that there was significant (~50%) prolongation of the left atrial APD_75_ in the atrial AMPK-dKO hearts (Figure 6F). Notably, this electrical remodeling was restricted to the atria, as the corresponding left ventricular APD_75_ was comparable in atrial AMPK-dKO and control hearts (Figure 6F). Thus, altered potassium channel expression was associated with prolonged atrial repolarization in AMPK-dKO mice.

### Atrial AMPK deletion decreases Pitx2 and Mef2c expression in the left atrium.

**Figure 7:**
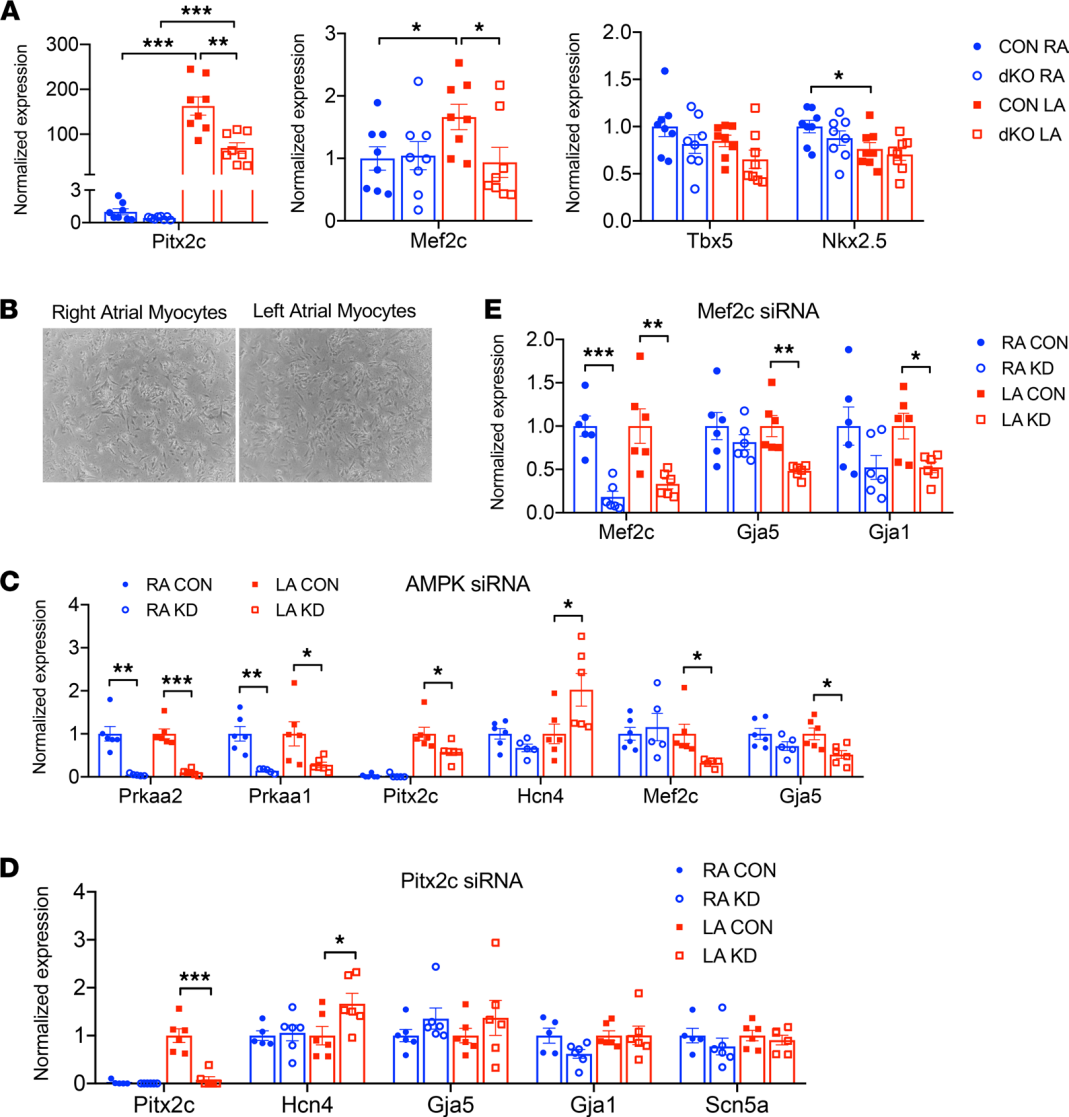
AMPK modulates the expression of the funny current channel and connexins in the left atrial cardiomyocytes through regulation of *Pitx2c* and *Mef2c*. Transcription factor expression in AMPK double-KO *sarcolipin-Cre Prkaa1^fl/fl^ Prkaa2^fl/fl^* (AMPK-dKO) and littermate control *Prkaa1^fl/fl^ Prkaa2^fl/fl^* (CON) mice. Effects of transcription factor knockdown in isolated left atrial (LA) and right atrial (RA) myocytes. (**A**) mRNA transcript levels of the transcription factors *Pitx2c*, *Mef2c* *Tbx5*, and *Nkx2.5* in right atria and left atria of 1-week-old mice. Values are mean ± SEM of *n* = 6. *P < 0.05, **P < 0.01, ***P < 0.001 versus CON by unpaired Student’s *t* test. (**B**) Representative micrographs of primary right and left atrial cardiomyocytes isolated from neonatal rats. (**C**) mRNA transcript levels of transcription factors, ion channels, and connexins after siRNA-mediated knockdown of AMPK α1 (*Prkaa1*) and AMPK α2 (*Prkaa2*) in right and left atrial cardiomyocytes. (**D**) mRNA transcript levels of channels and connexins after siRNA knockdown of *Pitx2c*. (**E**) mRNA transcript levels of connexin-40 (*Gja5*) and connexin-43 (*Gja1*) after *Mef2c* knockdown. Values are mean ± SEM of *n* = 6–8 per group. *P < 0.05, **P < 0.01, ***P < 0.001 versus scrambled siRNA controls by unpaired Student’s *t* test.

To investigate the upstream mechanisms underlying atrial electrical remodeling, we performed an unbiased gene profile analysis to detect genes that were altered in the atrial AMPK-dKO model. There were 439 atrial genes that were differentially regulated as compared with control mice, 158 upregulated and 271 downregulated. We focused the analysis on identifying alterations in transcriptional pathways that are known to play a role in left-right asymmetry, since we had noticed that several genes, including *Gja5* and *Kcnj2*, appeared to be differentially affected by AMPK depletion in the left compared with the right atrium. Microarray data (Supplemental Table 1) revealed an interesting reduction in *Pitx2*, which encodes a homeobox transcription factor that plays a pivotal role in left-right determination during embryonic development (24). We then performed real-time quantitative PCR (RT-qPCR), which demonstrated a 160-fold higher expression of *Pitx2c* in left atria compared with right atria in control mice, consistent with its predominant left atrial expression pattern after development. However, the atrial AMPK-dKO mice exhibited a 60% reduction in *Pitx2c* mRNA expression in the left atria, while its level in the right atria was unchanged, thereby reducing the interatrial *Pitx2c* gradient (Figure 7A).

The microarray screen also identified differential regulation of another transcription factor, *Mef2c*, known for its role in cardiac morphogenesis and myogenesis (25). RT-qPCR showed that *Mef2c* mRNA expression was 75% higher in the left atria compared with the right atria in control mice (Figure 7A). This interatrial gradient was abolished in the atrial AMPK-dKO mice because of selective downregulation of *Mef2c* in the left but not the right atria (Figure 7A). Other key transcription factors essential for cardiac development, such as *Tbx5* and *Nkx2-5*, were neither differentially expressed across the atria nor affected by AMPK deletion (Figure 7A).

### AMPK knockdown modulates levels of transcription factors that control the expression of ion channels and gap junction proteins in left atrial cardiomyocytes.

In order to more directly delineate the role of AMPK signaling in modulating transcriptional control of atrial ion channel subunits and gap junction proteins, we established an in vitro model of AMPK knockdown to avoid the possibility of compensatory genetic reprogramming in vivo. Since electrical remodeling was evident in the atrial AMPK-dKO mice as early as 1 week of age, we investigated the direct effects of AMPK knockdown in rat neonatal cardiomyocytes (Figure 7B), which achieved more efficient siRNA knockdown than could be accomplished in mouse atrial cardiomyocytes.

Using siRNAs targeted against *Prkaa1* (AMPK α1) and *Prkaa2* (AMPK α2), we observed greater than 75% efficiency of AMPK knockdown in separate right and left atrial cardiomyocyte preparations (Figure 7C). Silencing of AMPK in atrial cardiomyocytes led to a significant downregulation of *Pitx2c*, *Mef2c*, and *Gja5* while increasing *Hcn4* expression in the left atrial cardiomyocytes. Interestingly, there were no significant changes in right atrial cardiomyocytes despite their comparable degree of AMPK knockdown (Figure 7C). Overall, the results in isolated atrial cardiomyocytes paralleled our in vivo findings in the atrial AMPK-dKO mouse model. Further, these in vitro studies revealed greater intrinsic susceptibility of left atrial cardiomyocytes to molecular remodeling consequent to AMPK deletion than their right atrial counterparts.

To further understand the relationship between the transcription factors and the ion channels and connexins that were regulated by AMPK, we proceeded to knock down *Pitx2c* and *Mef2c* in the cardiomyocytes. Silencing *Pitx2c* resulted in upregulation of *Hcn4* expression in left but not right atrial cardiomyocytes, despite a comparable level of *Pitx2c* knockdown in the latter cells (Figure 7D). Silencing *Mef2c* resulted in significant reductions in *Gja1* and *Gja5* in left atrial cardiomyocytes, while their levels in right atrial cardiomyocytes were not significantly altered despite comparable *Mef2c* knockdown (Figure 7E). Potassium channel expression was not significantly affected by the knockdown of either *Pitx2c* or *Mef2c* in left or right atrial cardiomyocytes (Supplemental Figure 5). Taken together, these findings highlight potentially previously unrecognized molecular mechanisms mediating electrical remodeling in left atrial cardiomyocytes. They indicate that AMPK has an important role in regulating transcription factors, which control the expression of genes encoding proteins that are critical to atrial electrical function.

After completion of these studies, a different genotyping service instituted by our new animal care facility identified cases of heterozygous germline excision of *Prkaa1* and *Prkaa2* isoforms in our mouse colony. Therefore, we investigated the potential impact of such germline excision on the results. Heterozygous excision of *Prkaa1*, *Prkaa2*, or both had no effects on the atrial electrophysiological findings in control mice, since mice with heterozygous excision demonstrated similar heart rate variability to normal control mice (Sln Cre^–^ with homozygous floxed *Prkaa1* and *Prkaa2* alleles) (Supplemental Figure 6). In addition, heterozygous excision of *Prkaa1*, *Prkaa2*, or both was well-compensated for in left ventricular tissue; there was no evidence of reduced AMPK α1 or AMPK α2 expression or diminished phosphorylation of the AMPK downstream target ACC when comparing mice with heterozygous excision to normal control mice (Sln Cre^–^ with homozygous floxed *Prkaa1* and *Prkaa2* alleles) (Supplemental Figure 7). Outside of the heart, the liver showed a decrease in AMPK α1 expression but preserved AMPK α2 expression in the context of heterozygous *Prkaa1* and *Prkaa2* excision, respectively, but downstream ACC phosphorylation remained intact in liver samples from mice with heterozygous excision of *Prkaa1*, *Prkaa2*, or both.

## Discussion

The results of this study implicate the critical role of AMPK signaling in the governance of atrial homeostasis by defining the hierarchical alterations in electrophysiological function and structural remodeling caused by selective deletion of AMPK in the atria. Mice with conditional deletion of the AMPK α catalytic subunits in atrial myocytes developed atrial conduction and repolarization delays and atrial ectopic activity before the subsequent onset of spontaneous atrial fibrillation. Left atrial electrophysiological abnormalities could be explained, in part, by reprogramming of ion channels and connexins prior to the development of left atrial structural remodeling or fibrosis. The perpetuation of atrial fibrillation correlated temporally with the development of atrial interstitial fibrosis. The findings also provide insight into the molecular underpinnings of the atrial electrophysiological reprogramming, specifically revealing a link between AMPK deletion and the reduced expression of the left-sided transcription factors, *Pitx2c* and *Mef2c*. While *Pitx2c* exerted negative inhibitory effects on *Hcn4* expression, *Mef2c* positively regulated the expression of connexins.

### AMPK and atrial structural remodeling.

Atrial-specific AMPK deletion caused early right but not left atrial structural remodeling in mice prior to the onset of spontaneous sustained atrial fibrillation. Using contrast-enhanced micro-CT in mice as young as 4 weeks of age, we found early dilatation of the right atrium in the absence of right ventricular enlargement or contractile dysfunction, indicating that the atrial enlargement was a primary consequence of AMPK deletion. This differential right compared with left atrial enlargement points to chamber asymmetry in the biological response to the loss of AMPK activity. Interestingly, prominent right atrial enlargement was also reported in mice with striated muscle deletion of LKB1, which is the upstream kinase for AMPK and 12 other AMPK-related kinases (26). We later observed interstitial fibrosis in both the right and left atria at 8 weeks of age in the atrial AMPK-dKO mice, correlating with the onset of detectable atrial fibrillation. Subsequent left atrial dilatation was identified at 15 weeks of age, a time when most of the atrial AMPK-dKO mice demonstrated spontaneous atrial fibrillation. These latter findings suggest that left atrial remodeling could be a sequela of superimposed atrial fibrillation and/or the delayed consequence of AMPK deletion.

### AMPK and electrophysiological remodeling.

The electrocardiographic assessment of atrial AMPK-dKO mice demonstrated early prolongation of the P-wave duration in vivo, consistent with the delayed left atrial activation revealed by optical mapping of hearts ex vivo. In the absence of left atrial fibrosis or dilatation, these electrical abnormalities likely reflect downregulation of connexin and Nav1.5 expression, which occurred in this chamber prior to the onset of atrial fibrillation. The decrease in upstroke velocity observed in the left atrial optical action potential is also consistent with impaired conduction, likely due to reduced Nav1.5. Interestingly, a reduction in *Scn5a* encoding this channel was previously observed in mice with cardiac LKB1 deletion (27).

Atrial fibrillation is often associated with narrowing of the effective refractory period. However, prolongation of the refractory period can occur in a rate-dependent manner in the setting of underlying heart disease (28, 29). Such prolongation may contribute to atrial fibrillation by promoting arrhythmogenic triggers and increasing spatiotemporal repolarization gradients (5). AMPK deletion in our mouse model led to a prolongation of the left atrial action potential prior to the development of atrial fibrillation. This could be related in part to the associated downregulation of potassium channels that mediate repolarization of the atrial cardiomyocytes, including *Kcna5* and *Kcnq1*, which encode the Kv1.5 and Kv7.1 channels, respectively. Although the prolonged action potential duration may have acted to inhibit atrial fibrillation at an early age in the AMPK-dKO mice, reduced repolarizing currents might have been arrhythmogenic later in this model by inducing early atrial afterdepolarizations, action potential alternans, or the dispersion of repolarization (30).

AMPK deletion also led to the predominantly left-sided downregulation of *Kcnj2* transcripts and a diminished expression of the Kir2.1 protein that they encode. The Kir2.1 channel constitutes the main component of the inward rectifier potassium current, *I*_K1_ (31). In addition to its contribution to final repolarization, *I*_K1_ plays an important role in setting the resting membrane potential. As such, Kir2.1 downregulation would be expected to destabilize the resting membrane potential, which in combination with increased *Hcn4* expression in the left atrium could promote abnormal atrial automaticity and ectopy. The atrial AMPK-dKO mice demonstrated ectopic premature atrial complexes, which may also be triggers for atrial fibrillation, in contrast to the control mice that displayed uninterrupted normal sinus rhythm on the ECG. It is of interest to note that many of the ion channels and gap junction proteins that were altered by atrial AMPK deletion have been implicated in other murine models of atrial fibrillation.

### AMPK regulates transcription factors.

To our knowledge, these findings are the first to reveal that loss of AMPK affects the expression of cardiac transcription factors involved in right-left asymmetry and that these transcription factors regulate the expression of mRNA transcripts encoding gap junction proteins and ion channels in the atrium. Ion channels and connexins are known to be regulated via posttranslational phosphorylation by protein kinases, which modulate their activity and subcellular targeting (32–34). However, our results demonstrated a marked decrease in *Mef2c* transcripts, specifically in the left atria of the AMPK-dKO mice, and further showed the effect of AMPK knockdown to reduce *Mef2c* in primary left atrial myocytes. MEF2C is a cardiac-enriched transcription factor that is critical in heart development (35). Silencing *Mef2c* also resulted in reductions in connexin-40 expression in left atrial myocytes. Thus, these results implicate connexin-40 as a target of *Mef2c* and indicate a functional link between loss of AMPK and both MEF2C and connexin-40 expression in the left atrium.

Our studies also indicate that loss of AMPK activity affects atrial *Pitx2c* expression. In addition to the decrease in *Pitx2c* transcripts in the left atria of AMPK-dKO mice, in vitro knockdown of AMPK reduced *Pitx2c* expression in primary left atrial myocytes. PITX2 is a bicoid-related homeodomain transcription factor that has an essential role in directing cardiac asymmetric morphogenesis (24, 36). Although AMPK deletion had numerous effects on atrial gene expression, silencing *Pitx2c* recapitulated the effects of AMPK knockdown to increase *Hcn4* in primary left atrial cardiomyocytes. Prior studies have shown that mice deficient in PITX2C display right isomerism (37), suggesting that PITX2C inhibits the sinoatrial pacemaker activity in the left atrium (38, 39). Atrial deletion of PITX2 also causes atrial enlargement with extensive remodeling of ion channels, including *Scn5a* and *Kcnj2*, and conduction abnormalities (40). Thus, taken together, our findings indicate a functional link between metabolic signaling and transcriptional regulation, with AMPK deletion leading to loss of the inhibitory effect of *Pitx2c* on *Hcn4* expression.

### Temporal hierarchy and asymmetry of the atrial consequences of AMPK depletion.

Several observations should be highlighted regarding the sequence of pathogenic alterations in the atria consequent to AMPK deletion. Early electrophysiological remodeling, including prolongation of the P-wave duration and altered expression of channels responsible for atrial conduction and repolarization, was evident well before structural atrial remodeling. There was also asymmetric structural remodeling of the atria, with right atrial enlargement evident early prior to the onset of atrial fibrillation and left atrial enlargement developing several weeks later after the detection of atrial fibrillation. As noted, atrial fibrosis was detected at the stage that spontaneous atrial fibrillation started to emerge, consistent with the concept that fibrosis is important for the perpetuation of atrial arrhythmia (41). Despite its lesser early predilection to structural remodeling, the left atria of the atrial AMPK-dKO mice demonstrated more prominent alterations in the transcript levels of both channels (*Gja1*, *Gja5*, *Hcn4*, and *Kcnj2*) and transcription factors (*Pitx2c* and *Mef2c*) at a young age. These findings were recapitulated and even more pronounced in a cell-autonomous fashion, as evidenced by the differentially greater effects of siRNA knockdown of AMPK in isolated rat neonatal left compared with right atrial cardiomyocytes. In addition, transcription factor knockdown caused more dramatic alterations in channel expression in left compared with right atrial cardiomyocytes. The lesser effects of AMPK knockdown in isolated right atrial cardiomyocytes as compared with atrial tissue from the AMPK-dKO mice may reflect age or time dependence but also raise the interesting possibility that early stretch of the right atria in vivo might also have contributed to its channel remodeling in the intact mouse. Thus, the results overall highlight context-specific biological complexity in the response of the atria to metabolic stress, which is of interest and warrants additional investigation.

### Clinical implications.

We hypothesize that impaired AMPK activity may contribute to the high incidence of atrial fibrillation in elderly patients with metabolic diseases (4). Both aging and diabetes can cause impaired AMPK activation (42, 43), although the effects of these conditions on AMPK in the human heart are not known. Reduced AMPK phosphorylation was previously reported in atrial tissue excised during cardiac surgery from patients with persistent atrial fibrillation, while those with paroxysmal atrial fibrillation demonstrated increased AMPK phosphorylation (20). AMPK activation was further shown to protect against rapid pacing-induced metabolic stress in canine atrial cardiomyocytes (20). Together, these findings predict that a decrease in AMPK activity in susceptible patients may result in an electrophysiological reprogramming of atrial ion channels and gap junction proteins that might promote the development and perpetuation of atrial fibrillation.

AMPK deletion disrupted the expression of several genes encoding channels and transcription factors that have been linked to human atrial fibrillation. For instance, genome-wide association studies have reported risk variants adjacent to *PITX2* located on chromosome 4q25 that are strongly associated with atrial fibrillation (44). Mutations that result in the unstable expression of connexin-40 are also associated with lone atrial fibrillation (45). Disruptions in sodium influx via Nav1.5 channels have been correlated with an increased incidence of atrial fibrillation in humans (45, 46). Thus, intact AMPK function appears to have an important homeostatic role in the atria that affects downstream molecular mechanisms that have been implicated in human atrial fibrillation.

### Study limitations.

One limitation to note is that after completion of these studies, we identified examples of heterozygous germline deletion subsequently in our colony. Further investigation revealed that the mice compensated for heterozygous excision with no evidence of impaired AMPK downstream signaling. In addition, heterozygous excision of *Prkaa1*, *Prkaa2*, or both also had no effect on atrial electrophysiology. Thus, although we cannot fully exclude the possibility, it is unlikely that heterozygous background excision influenced the phenotype of either the AMPK-dKO or control mice.

### Conclusion.

In summary, AMPK plays a crucial role in maintaining electrophysiological homeostasis and preserving normal conduction in the atria. Atrial AMPK protects against the dysregulation of transcription factors that are essential for maintaining proper ion channel and gap junction protein expression. Deletion of AMPK in the atria promotes both the triggers and the substrate that predispose to the development of atrial fibrillation. These findings provide the rationale to investigate whether AMPK activators might have a role in the prevention and treatment of atrial fibrillation.

## Methods

### Generation of AMPK α1α2-KO mice.

We generated mice with an atrium-specific AMPK α1 α2 dKO by initially crossing *Prkaa1^fl/fl^* and *Prkaa2^fl/fl^* mice (47) on a C57BL/6 background (Charles River). Then, homozygous *Prkaa1^fl/fl^* *Prkaa2^fl/fl^* mice were crossed with C57BL/6 mice (48) expressing Cre-recombinase under the control of the Sln promoter (*Sln-Cre^+/–^*) to drive transcription in the atria. Mice expressing the Sln Cre-recombinase alone (*Sln-Cre^+/–^ Prkaa1^+/+^ Prkaa2^+/+^*) were used as additional controls. Mice were backcrossed in the C57BL/6 background for at least 7 generations.

After completion of these studies, this colony was relocated, and genotyping was performed by Transnetyx using RT-qPCR with primers to WT, floxed, and excised *Prkaa1* and *Prkaa2* alleles (Supplemental Table 2).

### ECG.

Surface ECGs were performed under isoflurane anesthesia (1%–2%) in an electrically shielded chamber to minimize background electrical noise interference, maintaining normal body temperature as previously described (27). Signal-averaged tracings of 10 consecutive beats were obtained using LabChart (AD Instruments), and at least 50 tracings per animal were quantified to calculate the P-wave duration, PR interval, QRS duration, and average heart rate. Spontaneous atrial fibrillation was defined as the presence of disorganized atrial activity with irregularly conducted QRS complexes, lasting more than 30 seconds in at least 2 separate 1-minute recordings on the same day. Heart rate variability was assessed during 60 seconds by comparing sequential R-R intervals (49).

### Transthoracic echocardiography.

Mice were anesthetized using isoflurane (1%–2%), and transthoracic echocardiography was performed while maintaining body temperature using a high-resolution ultrasound instrument (VisualSonics 2100) as previously outlined (27, 50).

### Contrast ECG-gated micro-CT.

Mice were anesthetized with isoflurane (1%–2%) and 80–100 μL of CT contrast (Exitron 12000, Miltenyi Biotec) was injected via a lateral tail vein. An ECG-gated CT scan was acquired on a hybrid SPECT/CT scanner (U-SPECT^4^CT MILabs). The ultra-focus CT system protocol (50 kVp, 0.48 mA) was used for assessment of atrial and ventricular chamber volumes and function. Images were reconstructed with filtered 8 back projection with a voxel size of 80 μm. The endocardial surfaces of the CT images were segmented from 3D images using semiautomated software (ITK-snap, http://www.itksnap.org) (51).

### Immunohistochemistry.

After mice had been heparinized and anesthetized with pentobarbital, the heart was perfused with 4% paraformaldehyde in cardioplegia solution as described previously (27).

### Hydroxyproline assay.

Whole atrial tissues were first hydrolyzed in 20% HCl at 120°C. After centrifuging the samples, the supernatants were transferred to a clear, uncoated, 96-well plate. Oxidized hydroxyproline was mixed with 4-(dimethylamino)benzaldehyde (Sigma-Aldrich) to produce a colorimetric product with its absorbance measured at 560 nm.

### RT-qPCR and immunoblot analysis.

Gene expression analyses were performed by RT-qPCR as previously described (27). Rapidly frozen right and left atrial tissues were homogenized using a bead mill TissueLyser II (Qiagen). Atrial lysates were prepared for immunoblotting using established methods (27). The following antibodies were used: AMPKα (D5A2), AMPKα2 (catalog 2757), phospho-AMPKα Thr^172^ (D4D6D), ACC (C83B10), phospho-ACC Ser^79^ (D7D11), connexin-43 (E7N2R), Nav1.5 (D9J7S), GAPDH (14C10) (all from Cell Signaling Technology), and Kir2.1 (N112B/1) (Abcam).

### Optical mapping of Langendorff-perfused murine hearts.

Hearts from AMPK-dKO and age-matched control mice were cannulated and retrogradely perfused through the aorta. Hearts were oriented within a custom-designed imaging chamber to ensure optimal exposure of the left atrium and left ventricle in the field of view. This orientation was chosen to elucidate the physiological consequences of the predominant molecular alterations that were more prominent in the left atrium. The optical mapping system was optimized for rodent hearts as previously described (52). Hearts were stained with the voltage-sensitive dye di-4-ANEPPS (Invitrogen) and exposed to a monochromatic excitation light. Emitted light was filtered through a long pass band filter (>630 m) and projected onto an 80 × 80 pixel charge-coupled device camera (SciMeasure Analytical Systems), yielding an interpixel spatial resolution of 50 μm. Volumetric ECG recordings (Biopac Systems MP150), as well as perfusion pressure and temperature, were recorded throughout the experiment. Mechanical contractions were minimized using an electromechanical uncoupler (Blebbistatin, Sigma-Aldrich). Hearts were paced from the left atrium at 10 Hz (pacing cycle length = 100 ms) using a unipolar electrode at 1.5× diastolic threshold. Atrioventricular capture was confirmed both electrically and optically.

### Analysis of periodic optical signals.

Raw data were collected, frame-selected, and processed in CardioPlex (RedShirt Imaging) and a custom-made software written in MATLAB (MathWorks). To improve signal-to-noise ratio, frames were temporally averaged over 8 consecutive beats. Images were further spatially averaged using a 5 × 5 uniform kernel at each pixel, normalized, baseline subtracted, and inverted. Activation delays were determined across left atrial and left ventricular mapping fields during steady-state pacing. Action potential duration was calculated for each pixel as the time difference between the action potential upstroke and the point at 75% repolarization from the peak (APD_75_). The values for the left atrium and ventricle represent the average of all action potential durations within the region of interest.

### Atrial myocyte isolation.

Neonatal atrial cardiomyocytes were isolated from rats (P1–P3) using collagenase/dispase-containing L15 medium (53). Suspended cardiomyocytes were plated into cell culture dishes precoated with collagen and were left undisturbed in the incubator for 18 hours to allow for adherence and spreading of cardiomyocytes.

### Cell transfection assays.

Mouse atrial cardiomyocytes underwent knockdown of *Prkaa1*, *Prkaa2*, *Pitx2c*, and *Mef2c* by siRNA treatment (Qiagen). Transfection of cells with siRNA was performed using a standard protocol with Lipofectamine RNAiMAX (Invitrogen).

### Data availability.

The data sets generated and/or analyzed during the current study are available from the corresponding author on reasonable request. Gene expression data files are available (National Center for Biotechnology Information’s Gene Expression Omnibus GSE200144).

### Statistics.

Results are presented as mean ± SEM. Statistical analyses were performed using GraphPad Prism 6. Comparisons between groups were made by parametric and nonparametric tests, including 2-tailed Student’s *t* test and 2-way ANOVA. For serial comparison of the echocardiogram and CT results, 2-way ANOVA and Holm-Šidák multiple-comparison test were used. A *P* value of less than 0.05 was considered statistically significant.

### Study approval.

All protocols involving mice were approved by the Yale IACUC.

## Author contributions

KNS designed research studies, conducted experiments, acquired and analyzed data, and contributed to writing the manuscript. YM designed research studies, analyzed data, and contributed to writing the manuscript. MC, ZI, NR, XW, and NG conducted experiments and acquired and analyzed data. GKM designed research studies, analyzed data, and contributed to writing the manuscript. SLT conducted experiments, acquired and analyzed data, and contributed to writing the manuscript. AJS designed research studies, analyzed data, and contributed to writing the manuscript. MF and BV provided reagents and contributed to writing the manuscript. JGA analyzed data and contributed to writing the manuscript. FGA and LHY designed research studies, analyzed data, and contributed to writing the manuscript.

## Supplementary Material

Supplemental data

Supplemental table 1

## Version 1 04/22/2022

Electronic publication

## Version 2 05/06/2022

Updated issue number
