# Supplementary material for: Atrial AMP-activated protein kinase is critical for prevention of dysregulation of electrical excitability and atrial fibrillation
Source: JCI Insight. 2022 Apr 22;7(8):e141213. doi: 10.1172/jci.insight.141213 (PMC9089788; doi:10.1172/jci.insight.141213)
Supplement: Supplemental data [file jciinsight-7-141213-s181.pdf]

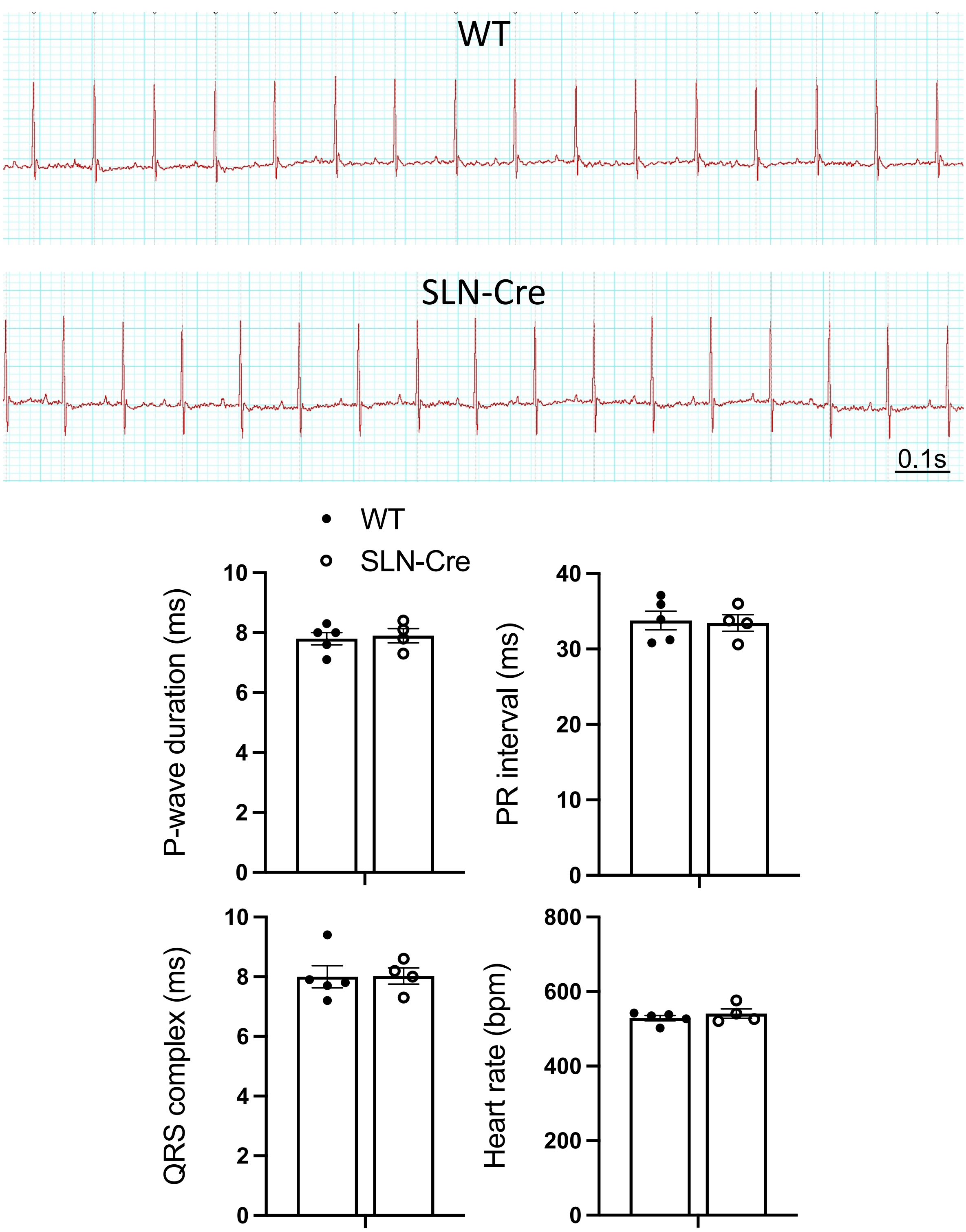

Supplemental Figure 1. Electrocardiographic findings in sarcolipin-Cre AMPK $\alpha1^{+/+}\alpha2^{+/+}$ (SLN-Cre) compared to wild type (WT) mice at 8 weeks of age. Representative ECG tracings in the upper panel and quantification of ECG parameters in lower panel.

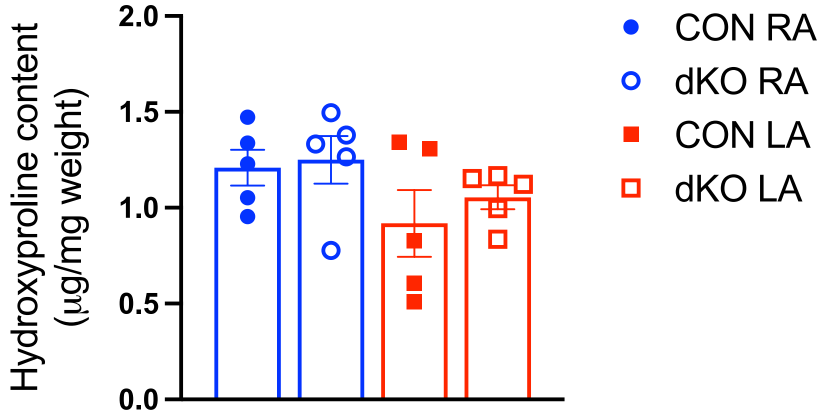

Supplemental Figure 2. Hydroxyproline content in right atrial (RA) and left atrial (LA) myocardial homogenates from control (CON) and atrial AMPK double knockout (dKO) mice at 4 weeks of age.

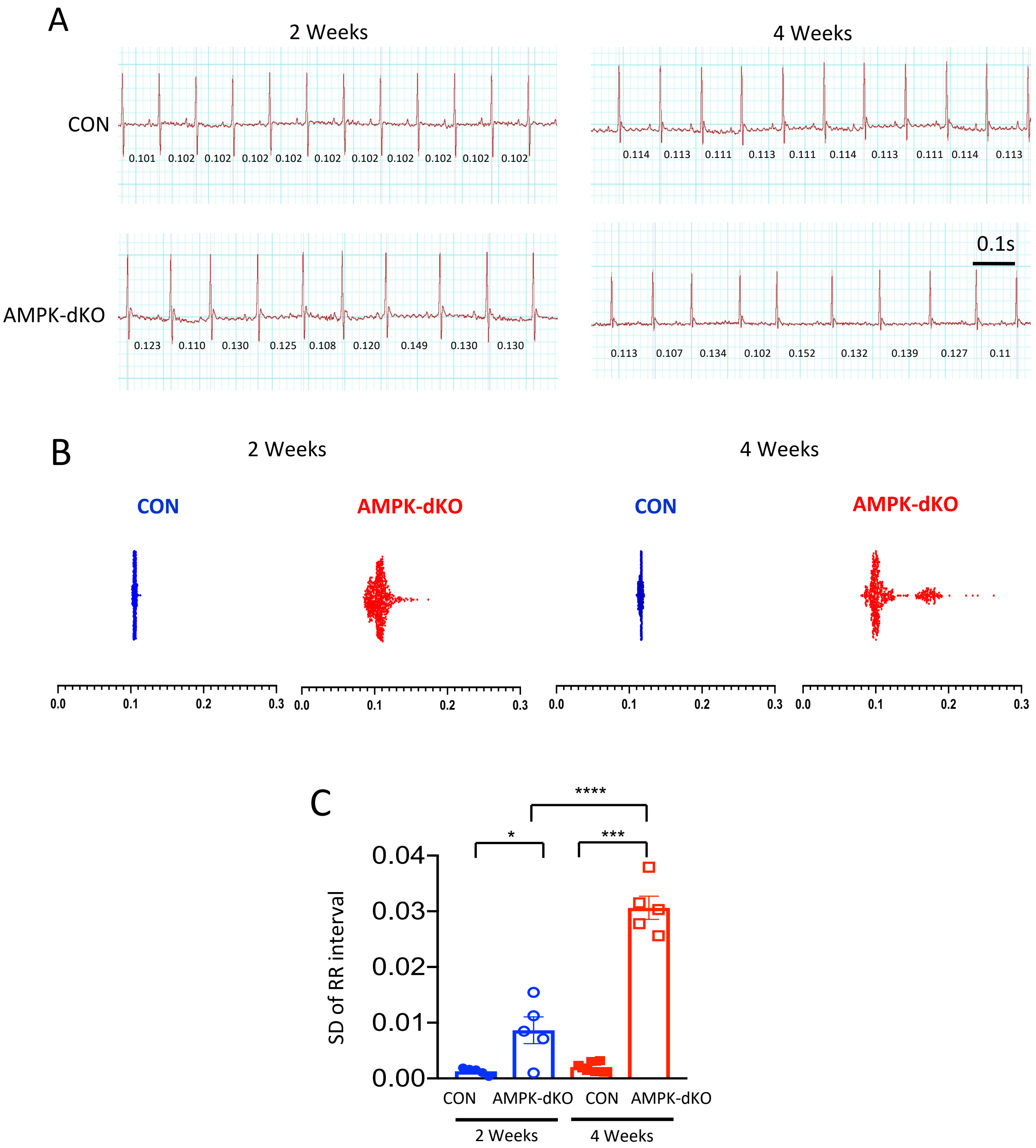

Supplemental Figure 3. Atrial ectopy in atrial AMPK double knockout (dKO) mice mice. (A) Representative examples of electrocardiogram tracings in control (CON) and AMPK-dKO mice at 2 and 4 weeks of age including individual RR intervals (sec). (B) Representative graphs of the variability of individual RR intervals in the CON and AMPK-dKO mice during 1-minute recordings under light inhaled anesthesia. (C) Comparison of standard deviations (SD) in the heart rate, quantified during the 1-minute recordings in AMPK-dKO and CON mouse (n=5 per group). *p<0.05, ***p<0.001 vs. CON; ****p<0.0005 at 2 vs. 4 weeks of age.

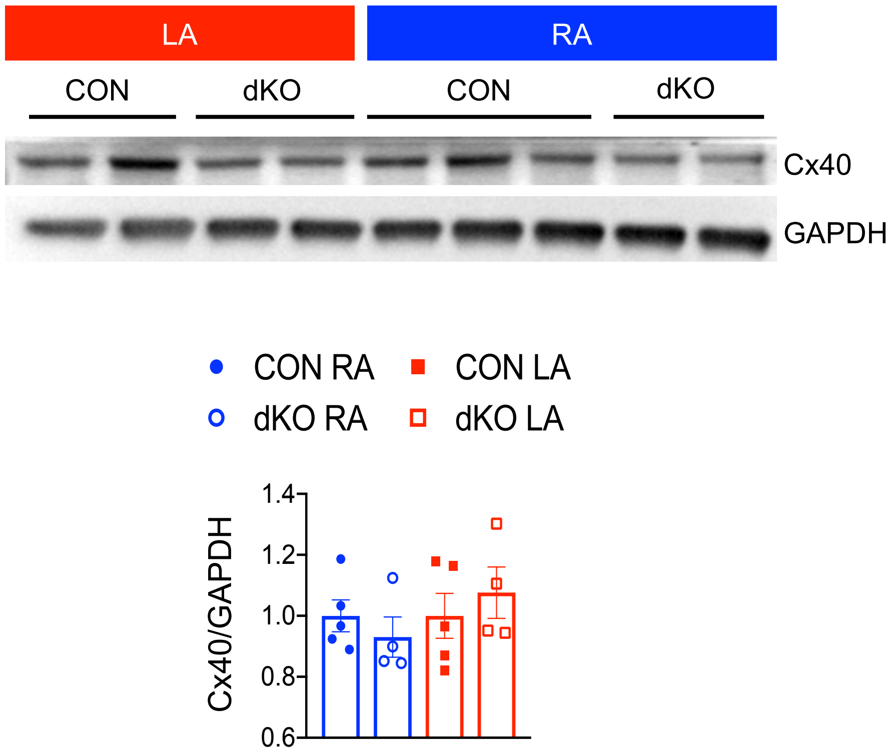

Supplemental Figure 4. Connexin 40 (Cx40) immunoblots showing its expression in the left atria (LA) and right atria (RA) of atrial AMPK double knockout (dKO) and control (CON) mice at 4 weeks of age.

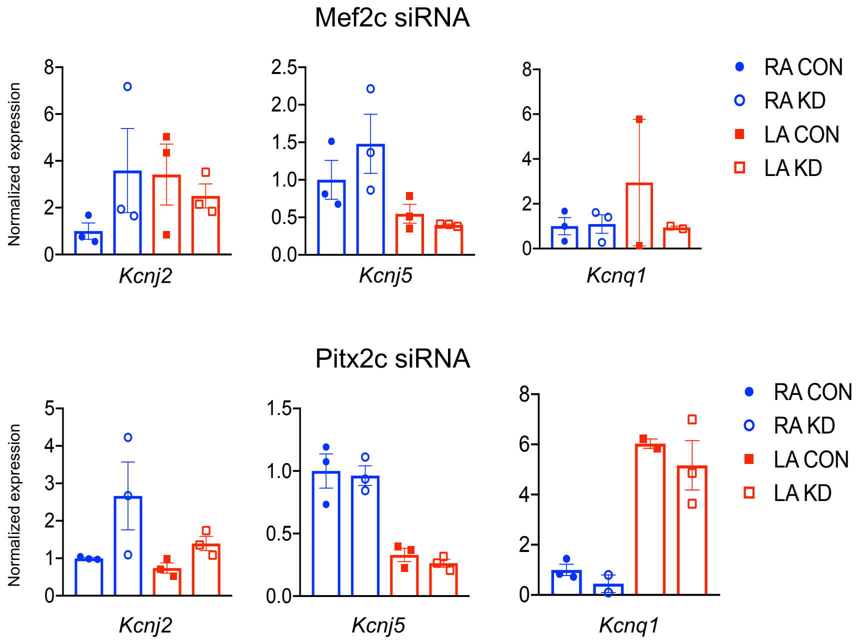

Supplemental Figure 5. Potassium channel expression after knockdown of the transcription factors Mef2c and Pitx2c in atrial cardiomyocytes. The mRNA transcript levels of *Kcnj2*, *Kcnj5*, and *Kcnq1* were measured in left and right atrial rat neonatal cardiomyocytes following siRNA-mediated knockdown (KD) of *Mef2c* (upper panel) and *Pitx2c* (lower panel).

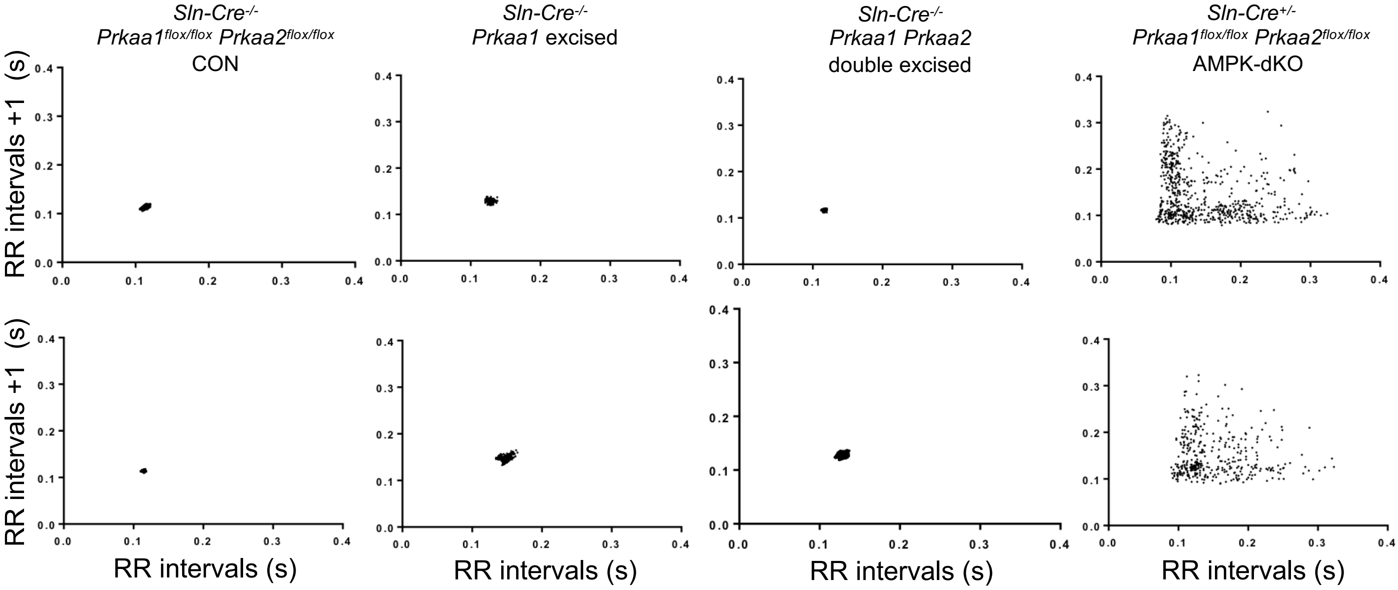

Supplemental Figure 6. Heart rate variability of a representative homozygous floxed *Prkaa1* and *Prkaa2* control (CON), heterozygous excision of *Prkaa1*, heterozygous excision of *Prkaa1* and *Prkaa2*, and AMPK double knockout (dKO). Graphs compare sequential R-R intervals during 60-second electrocardiographic recordings.

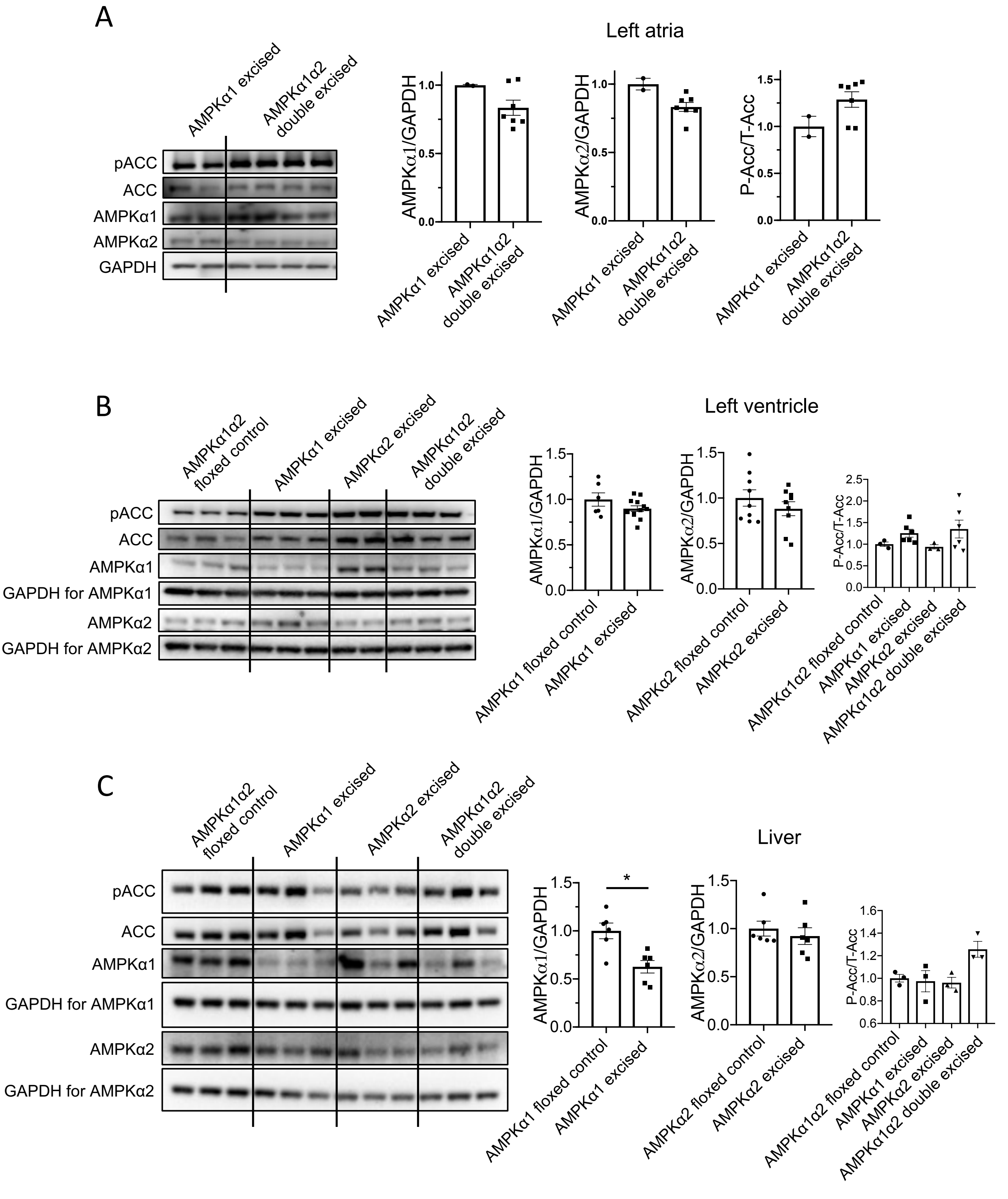

Supplemental Figure 7. Representative immunoblots with antibodies recognizing the α1 and α2 subunits of AMPK, the downstream AMPK target ACC, and the phosphorylated form of ACC (pACC) in the (A) left atria, (B) left ventricle, and (C) liver with corresponding densitometric quantification. GAPDH was used as loading control. Lysates were run on parallel gels simultaneously and probed for total proteins.

***Prkaa1* WT:**	
Forward Primer Sequence:	GCCCTGCTTGACCTCCAA
Reverse Primer Sequence:	AGAACAAGCAGAGACTGAGAAGGA
***Prkaa1* Floxed:**	
Forward Primer Sequence:	CCTGAATTTGCCCTGCTTGAC
Reverse Primer Sequence:	CGGCCGCGAAGTTCCT
***Prkaa1* Excised:**	
Forward Primer Sequence:	TCCAGAAGAAGATGTGTTTTTAAGTGTAGTT
Reverse Primer Sequence:	AGAGACTGAGAAGGAGCCAGATTA

***Prkaa2* WT:**	
Forward Primer Sequence:	CACTGAAATACATAGCAATTTCCAAACCTCAA
Reverse Primer Sequence:	CTGCGTTCCAAATGTATGCACC
***Prkaa2* Floxed:**	
Forward Primer Sequence:	TGTCTGCTTCTGCGTTCCAAAT
Reverse Primer Sequence:	GCCAGGCAGATGGTGCAT
***Prkaa2* Excised:**	
Forward Primer Sequence:	GCTTCTGCGTTCCAAATGCA
Reverse Primer Sequence:	GAATTCGATGGCCGCTCTAGATA

Supplemental Table 2. Real-time PCR primer sequences to wildtype (WT), floxed, and excised *Prkaa1* and *Prkaa2* alleles.
